# Osteoporosis treatment gap and prescribing patterns in Ireland: a cross-sectional analysis of the DXA HIP project

**DOI:** 10.1136/bmjopen-2025-107028

**Published:** 2026-03-09

**Authors:** John J Carey, Attracta Brennan, Catherine Armstrong, Fiona Heaney, Aoife Dempsey, Rebecca Egan, Kelly Gorham, Lan Yang, DXA MAP, E Erjiang

**Affiliations:** 1 Rheumatology, Galway University Hospitals, Galway, Ireland; 2 School of Medicine, College of Medicine, Nursing, & Health Sciences, University of Galway, Galway, Ireland; 3 School of Computer Science, College of Science & Engineering, University of Galway, Galway, Ireland

**Keywords:** Ireland, Primary Health Care, Fractures, Bone, RHEUMATOLOGY, Prescriptions

## Abstract

**Abstract:**

**Objectives:**

Previous studies suggest Ireland has the smallest osteoporosis treatment in Europe and very little inappropriate prescribing, in contrast to our experience. In this study, we examine the osteoporosis treatment gap in Ireland by assessing the prevalence of appropriate and inappropriate prescribing in 2 subgroups of the Irish dual-energy X-ray absorptiometry (DXA) Health Informatics Prediction (HIP) Project. Treatment eligibility was defined using established intervention thresholds, including prior fracture, femoral-neck T-score ≤−2.5, glucocorticoid use, or Fracture Risk Assessment Tool (FRAX) major osteoporotic fracture risk ≥20% or hip fracture risk ≥3%.

**Design:**

Secondary cross-sectional analysis of a subgroup of the DXA HIP Project Cohort.

**Setting:**

3 hospitals in the West of Ireland. DXA referrals come from primary care providers, hospital consultants and the osteoporosis service.

**Participants:**

5564 participants of a previously described convenience cohort including: (i) 3474 subjects referred for a DXA scan, and (ii) 2090 patients who completed a DXA scan.

**Results:**

82.4% were female with a mean age of 66.6 years, 59.6% of whom had a prior fracture. Prescribing data of calcium and vitamin D were available for 3738 (67.2%) subjects, and osteoporosis medication for 4157 (74.7%) subjects. Prescribing information was available for more than 99% of the DXA group, but just over 50% of the referral group. When examined in aggregate, the treatment gap is 6% for calcium and vitamin D and 38% for osteoporosis medication, in line with prior publications. However, among those with prescribing information and at least one indication for treatment, only 58.3% were prescribed calcium and vitamin D and 39.1% an osteoporosis medication. Furthermore, among patients without a clear indication for treatment, 50.6% were prescribed calcium and vitamin D, and 32.5% an osteoporosis medication.

**Conclusions:**

These data suggest the majority of patients with osteoporosis or at high risk of fracture in Ireland today do not receive appropriate osteoporosis treatment, while inappropriate prescribing is substantial. These findings suggest that the true treatment gap in Ireland is substantially larger than aggregate estimates imply.

**STRENGTHS AND LIMITATIONS OF THIS STUDY**

- The study uses a large dataset drawn from three hospitals providing dual-energy X-ray absorptiometry services across primary and secondary care.
- Standardised, internationally recognised thresholds were applied to classify treatment eligibility.
- Missing prescribing data in referral records reduces precision for some estimates.
- The cross-sectional design does not capture treatment adherence or longitudinal change.

## Introduction

 Performance metrics reflect acceptable standards of care that are implementable in clinical practice. Appropriate metrics are evidence-based, measurable and help assess the quality of care provided. These can help identify gaps in the care provided, aid in the development of effective implementation strategies for quality improvement and fiscal management to optimise value.^1^,^3^ In healthcare, value has been defined as the ratio of outcomes to long-term,^4^ and so the challenge is to reduce care which has no benefit and provide care which is both clinically and economically effective.^5^

Osteoporosis and the related fractures represent one of the most prevalent and devastating diseases worldwide today, with significant impacts on patients, healthcare systems and economies.^6 7^ A report on 29 European countries notes more than 4 million osteoporotic fractures (OF) occurred in 2019, accounting for almost €60 billion in healthcare costs.^7 8^ OF resulted in more than ¼ million deaths in 2019, and were the third leading cause of death in Sweden.^7^ However, osteoporosis lags behind many non-communicable diseases in terms of recognition and a clear understanding of the implications. This has resulted in a care gap which remains notably and lamentably large, in contrast to other non-communicable diseases such as cardiovascular disease.^7^ Among them, the treatment gap, which reflects the difference between the proportion of people who should be treated and the actual number of those who are, remains stubbornly large. ^2 7 9^This difference between recommended practice in osteoporosis care and what is actually taking place in practice has been described as a ‘chasm’.^3^

Reports suggest Ireland has one of the largest illness burdens related to osteoporosis in Europe, and projected to have the greatest increases over the coming decade.^7^ This is despite suggestions Ireland has the smallest treatment gap in Europe at 32%,^7^ and a lower rate of inappropriate prescribing and prescribing omissions for osteoporosis treatment than other European countries.^10^ This contrasts with our experience of providing osteoporosis and dual-energy X-ray absorptiometry (DXA) services for patients in the West of Ireland for the past two decades. In this study, we examined 2 sub-groups of the DXA Health Informatics Prediction (HIP) Project to assess the prevalence of appropriate and inappropriate osteoporosis prescribing: (i) 3474 patients referred for a DXA scan, and (ii) 2090 who completed a DXA scan and underwent medication assessment at the time of testing.

## Methods

### Patient and public involvement

Patients and members of the public were involved in the design and dissemination plans of our research.

### Study population

A cross-sectional analysis was performed of two subgroups of a previously described convenience cohort: The DXA HIP Project. This cohort represents individuals referred for DXA based on clinical indications. It reflects the population typically evaluated for osteoporosis risk in routine clinical practice. Following ethical approval, data were collected, merged, cleaned and de-identified from three hospitals in the West of Ireland including Galway University Hospital Merlin Park, Sligo University Hospital and Our Lady’s Hospital Manorhamilton. The details of the process and characteristics of the population have previously been described.^11 12^ A subsequent secondary analysis of data examining the prevalence of vertebral fractures and Fracture Risk Assessment Tool (FRAX) discordance was included in the current study to examine treatment rates and prescribing practices for the current study.^13 14^ FRAX scores were calculated using femoral-neck bone mineral density (BMD), consistent with FRAX recommendations.

We also examined referral and prescribing patterns among patients referred for a DXA scan in 2023 and early 2024 from Galway University Hospital. DXA scans are performed on patients referred by their general practitioner (GP), hospital consultant (HC) and our osteoporosis service (OS). Referral data are stored electronically and available for audit. GP referrals generally contain additional details such as height, weight, blood pressure, comorbidities and prescribed medications. Among 2773 referrals in 2023, 330 (12%) were rejected as either inappropriate or without justification; only accepted referrals are included in these analyses. Details of DXA scans at other facilities or our DXA centre were not included if not on the referral.

In Ireland, calcium and vitamin D are available for purchase with or without a prescription, while those with a medical card can obtain this with no fee when prescribed by their GP. Osteoporosis medication requires a prescription from either their GP or HC. Information on prescribed calcium and vitamin D and osteoporosis medication included on DXA referrals was available for this study. When attending for a DXA scan, our International Society for Clinical Densitometry (ISCD) trained and certified nurses perform a brief history, taking particular note of previous fracture details and osteoporosis medication use. If there is a discrepancy or uncertainty, they call the patients’ pharmacy to obtain current information to ensure our DXA report to the referring doctor contains current and accurate prescription information. Only femoral-neck T-scores were available in this dataset. Lumbar spine BMD measurements were not recorded, and therefore, individuals with osteoporosis isolated to the spine could not be identified. We only included the femoral-neck T-score for those with a DXA scan as this was the metric used to calculate the FRAX Ireland estimate and is the site recommended for men and women by the International Osteoporosis Foundation (IOF).^6^ FRAX scores were calculated only for individuals who were not receiving osteoporosis medication at the time of referral or DXA assessment, as FRAX is not validated for patients already on treatment.

### Outcomes

We compared the rates of appropriate prescribing of osteoporosis medication for those with at least one of the following commonly agreed intervention thresholds^15^,^17^:

A prior fragility fracture;

A femoral-neck T-score of ≤−2.5 for peri/postmenopausal women and men aged ≥50 years;

Glucocorticoid use;

A FRAX estimated risk of major osteoporotic fracture of ≥20% or hip fracture ≥3%;

Treatment eligibility was defined as meeting at least one of the above criteria: prior fracture, femoral-neck T-score ≤–2.5, glucocorticoid use, or a FRAX major osteoporotic fracture risk ≥20% or hip fracture risk ≥3%.

We also evaluated the rate of inappropriate prescribing of calcium and vitamin D, and osteoporosis medications for those without such an indication for treatment. Individuals meeting any of the above thresholds were classified as treatment-eligible. All others were classified as treatment-ineligible. Dietary calcium intake questionnaires and biochemical tests for vitamin D, calcium, phosphate and parathyroid hormone (PTH) are performed in outpatient clinics to help determine the need for such supplements but not available for these cohorts from the DXA registry. In clinical practice, we find the majority of those taking supplements do not need them, while many of those who do are not.

### Statistical analysis

Study participants were classified into treatment-eligible and treatment-ineligible groups based on established international guidelines using T-score thresholds, fracture history, FRAX scores and glucocorticoid use. Eligibility was determined separately for calcium/vitamin D supplementation and osteoporosis treatment. Descriptive statistics were used to summarise demographic and clinical characteristics, including age, gender and prevalence of key clinical risk factors. Treatment status for calcium/vitamin D and osteoporosis medication was assessed and compared against treatment eligibility. The proportion of individuals prescribed treatment despite not meeting criteria (over-treatment), and those meeting criteria but not treated (under-treatment), was calculated. One-proportion Z-tests were used to evaluate whether the proportion of treated vs untreated individuals significantly differed within each risk category and for each clinical indication (eg, prior fracture, glucocorticoid use, FRAX score thresholds). The analyses were descriptive and did not constitute a reclassification analysis. One-proportion Z-tests were used solely to compare the proportion prescribed treatment vs not prescribed treatment within each eligibility criterion. Analyses were conducted separately for the referral and DXA groups. All statistical analyses were performed using standard software, with a significance level set at p<0.05.

## Results

### Population characteristics

Data were available for 5564 subjects to be included in this study, summarised in table 1. 4585 (82.4%) are female with a mean age of 66.6 years, the majority of whom (59.6%) had a previous fracture. 29.7% are glucocorticoid users, while 40.5% had a previous DXA scan. These characteristics reflect the inherent referral bias in our DXA population since 52.7% come from HC and OS, the remainder (47.3%) from GP.

**Table 1:** Summary statistics of study subjects

Variable		Referral group	DXA group
N		3474	2090
Gender	N	3473	2090
	Female: N (%)	2895 (83.4)	1690 (80.9)
	Male: N (%)	578 (16.6)	400 (19.1)
Age in years	N	3474	2090
	Mean±SD	65.4±13.3	68.6±11.2
Height in metres	N	1009	2087
	Mean±SD	1.6±0.1	1.6±0.1
Weight in kg	N	924	2087
	Mean±SD	72.1±17.7	72.4±16.4
BMI in kg/m^2^	N	831	2087
	Mean±SD	27.2±5.9	27.6±5.6
Smoker	N	1297	1412
	No: N (%)	894 (68.9)	1200 (85.0)
	Yes: N (%)	403 (31.1)	212 (15.0)
Alcohol	N	914	1336
	No: N (%)	817 (89.4)	1316 (98.5)
	Yes: N (%)	97 (10.6)	20 (1.5)
Family history of osteoporosis	N	466	1090
	No: N (%)	247 (53.0)	862 (79.1)
	Yes: N (%)	219 (47.0)	228 (20.9)
Rheumatoid arthritis	N	704	1440
	No: N (%)	583 (82.8)	1180 (81.9)
	Yes: N (%)	121 (17.2)	260 (18.1)
Secondary osteoporosis	N	1387	1629
	No: N (%)	416 (30.0)	860 (52.8)
	Yes: N (%)	971 (70.0)	769 (47.2)
Repeat scan	N	3419	756
	No: N (%)	1991 (58.2)	495 (65.5)
	Yes: N (%)	1428 (41.8)	261 (34.5)
Prior fracture	N	1872	2088
	No: N (%)	656 (35.0)	944 (45.2)
	Yes: N (%)	1216 (65.0)	1144 (54.8)
Glucocorticoids use	N	1618	1501
	No: N (%)	1119 (69.1)	1073 (71.5)
	Yes: N (%)	499 (30.8)	428 (28.5)
Femoral-neck T-score	N	283	2006
	Mean±SD	−0.6±1.1	−1.5±1.0
FRAX MOF	N	30	1254
	Mean±SD	13.0±7.9	12.9±8.3
FRAX HF	N	23	1254
	Mean±SD	4.7±5.2	4.4±5.3
Calcium and/or vitamin D	N	1659	2079
	No: N (%)	875 (52.7)	802 (38.6)
	Yes: N (%)	784 (47.3)	1277 (61.4)
Osteoporosis medication	N	2073	2084
	No: N (%)	1198 (57.8)	1450 (69.6)
	Yes: N (%)	875 (42.2)	634 (30.4)

BMI, body mass index; DXA, dual-energy X-ray absorptiometry; FRAX, Fracture Risk Assessment Tool; HF, hip fracture; MOF, major osteoporotic fracture.

Data for age and gender were available for all but one patient, shown in table 1. Details of a prior fracture, whether the referral is for a new or repeat scan, and treatment are available for the majority of patients. GP referrals provide more information, the majority of which include height, weight, smoking, alcohol use, other diagnoses and medication (data not shown). In contrast, most hospital referrals include little information other than the patients name, age, gender and indication for a DXA scan. Interestingly, less than 1% of all referrals included a FRAX score or other fracture risk estimate.

Information on osteoporosis medication (99%) and current use of calcium and vitamin D (99%) are available for almost every patient who had a DXA scan. In contrast, these details are missing for many referral patients: 59.7% and 47.8% respectively. Among those receiving osteoporosis treatment, denosumab was the most frequently prescribed medication in both the referral (32.2%) and DXA (47.2%) groups. Oestrogen therapy was the next most frequent among the referral group (24.3%), followed by oral bisphosphonates (23.4%), teriparatide (7%) and zoledronate (5%). Only 1.5% were taking combination therapy. Among those who had a DXA scan, oral bisphosphonates were the second most frequently prescribed medication (36%), followed by zoledronate (5.4%) and teriparatide (4.7%), while only 1.9% were taking a combination of medication.

The referral and DXA groups were analysed separately because prescribing information was missing in up to 59.7% of referrals but was almost complete in the DXA group, where medication details were verified at the time of scanning. Analysing the groups separately reduces misclassification resulting from differences in data completeness.

### Treatment rates

When we examined prescribing data in aggregate, the number of subjects prescribed calcium and vitamin D (n=2061) is similar to number of subjects with an indication for treatment (n=2186), suggesting a treatment gap of 5.7%. Similarly, an aggregate assessment of osteoporosis medication prescribing suggests the majority of those with one or more indications for treatment (n=2419) are receiving an osteoporosis medication (n=1509), reflecting a treatment gap of 37.6%. However, when we examine the data by treatment-eligible or treatment-ineligible, it is apparent there is a large treatment gap. Among those with at least 1 indication for treatment, 41.7% were not prescribed calcium and vitamin D, while the majority, 60.9%, were not prescribed an osteoporosis medication. Of further concern, many patients prescribed calcium and vitamin D (50.6%) or osteoporosis medication (32.5%) had no clear indication for treatment, reflecting a significant amount of over-treatment. Further analyses, shown in tables 2–5, reveal less than 1 in 2 referred patients with an indication for treatment are prescribed an osteoporosis medication, while more than 1 in 3 without a clear indication are prescribed such treatment. Of further concern when examining the DXA group where there is almost complete medication information available, only 1 in 3 is prescribed appropriate treatment, though somewhat reassuringly only 1 in 6 was perhaps prescribed medication inappropriately. In clinical practice, we are seeing these patterns all the time whereby appropriate and inappropriate prescribing is very common. We do not have these data to include in these analyses. Online supplemental figures 1 and 2 illustrate the flow through the eligibility and treatment classification process.

**Table 2:** Calcium and/or vitamin D of referral group

Indication for treatment	N	Yes (%)	No (%)	P value*
Prior fracture	435	204 (46.9)	231 (53.1)	0.195
Glucocorticoids use	185	86 (46.5)	99 (53.5)	0.337
Femoral-neck T-score ≤−2.5	17	10 (58.8)	7 (41.2)	0.459
FRAX MOF ≥20% or HF ≥3%	13	8 (61.5)	5 (38.5)	0.392
Any indication	610	288 (47.2)	322 (52.8)	0.167
No indication	1049	496 (47.3)	553 (52.7)	0.077

* One-Proportion Z-Test is used to determine if the proportion of Yes differ from the proportion of No. P values in tables3^5^ used the same test.

FRAX, Fracture Risk Assessment Tool; HF, hip fracture; MOF, major osteoporotic fracture.

**Table 3:** Osteoporosis medication of referral group

Indication for treatment	N	Yes (%)	No (%)	P value
Prior fracture	637	321 (50.4)	316 (49.6)	0.842
Glucocorticoids use	221	85 (38.5)	136 (61.5)	<0.001
Femoral-neck T-score ≤−2.5	23	19 (82.6)	4 (17.4)	<0.001
FRAX MOF ≥20% or HF ≥3%	13	7 (53.8)	6 (46.2)	0.780
Any indication	839	398 (47.4)	441 (52.6)	0.137
No indication	1234	477 (38.7)	757 (61.3)	<0.001

FRAX, Fracture Risk Assessment Tool; HF, hip fracture; MOF, major osteoporotic fracture.

**Table 4:** Calcium and/or vitamin D of DXA group

Indication for treatment	N	Yes (%)	No (%)	P value
Prior fracture	1140	712 (62.5)	428 (37.5)	<0.001
Glucocorticoids use	427	274 (64.2)	153 (35.8)	<0.001
Femoral-neck T-score ≤−2.5	295	185 (62.7)	110 (37.3)	<0.001
FRAX MOF ≥20% or HF ≥3%	602	401 (66.6)	201 (33.4)	<0.001
Any indication	1576	987 (62.6)	589 (37.4)	<0.001
No indication	503	290 (57.7)	213 (42.3)	<0.001

DXA, dual-energy X-ray absorptiometry; FRAX, Fracture Risk Assessment Tool; HF, hip fracture; MOF, major osteoporotic fracture.

**Table 5:** Osteoporosis medication of DXA group

Indication for treatment	N	Yes (%)	No (%)	P value
Prior fracture	1144	420 (36.7)	724 (63.3)	<0.001
Glucocorticoids use	428	147 (34.3)	281 (65.7)	<0.001
Femoral-neck T-score ≤−2.5	296	117 (39.5)	179 (60.5)	<0.001
FRAX MOF ≥20% or HF ≥3%	605	234 (38.7)	371 (61.3)	<0.001
Any indication	1580	547 (34.6)	1033 (65.4)	<0.001
No indication	504	87 (17.3)	417 (82.7)	<0.001

DXA, dual-energy X-ray absorptiometry; FRAX, Fracture Risk Assessment Tool; HF, hip fracture; MOF, major osteoporotic fracture.

## Discussion

### Key Results

Here, we show the pattern of osteoporosis treatment prescribing is very concerning among a large cohort of ‘real-world’ subjects with and without osteoporosis or a high fracture risk in Ireland. Our results highlight widespread use of calcium and vitamin D for many patients whether they have an indication for treatment or not. In contrast, we found most of those with a clear indication for treatment are not taking an osteoporosis medication, while a significant proportion of those with no indication are. Taken together, these data show the treatment gap for osteoporosis in Ireland remains unacceptably large and reflects what we are seeing in practice. These data suggest prior reports suggesting the treatment gap and the rate of inappropriate prescribing in Ireland are small compared with other European countries represent an ecologic fallacy.

### Interpretation

Osteoporosis can be a devastating disease for those with severe or multiple fractures. Approved osteoporosis medications are generally safe and well tolerated, and can effectively reduce the risk of fracture.^18^ This is particularly true for postmenopausal women with a DXA diagnosis of osteoporosis or severe osteoporosis, a previous hip or spine fracture and women taking chronic glucocorticoid therapy.^6^,^815 16^ Several treatments are available in Ireland (alendronate, risedronate, ibandronate, zoledronate, denosumab, raloxifene, oestradiol, teriparatide, romosozumab), with differences in efficacy, side effects, route of administration and cost.^19^ These factors need consideration when addressing an individual’s unique situation and concerns. European reports suggest Ireland has one of the highest illness burdens related to osteoporosis and will have the greatest increase in fracture incidence and cost of care.^7 8^ Yet it is not a national priority, we have no national programme or specialist training for HC or GP, have no national screening programme, yet apparently have one of the smallest treatment gaps and rates of inappropriate prescribing in Europe.^710 20^,^23^ Even our own healthcare system suggests the estimated cost of treating osteoporotic fractures will increase from €1 billion in 2020 to €2 billion by 2030,^24^ yet there has been stubborn refusal to engage with experts and best practice standards for osteoporosis care.

A recent European report suggested the treatment gap for postmenopausal women in Ireland was the lowest of 29 countries at 32%, compared with a mean of 71%.^7^ Several Irish studies previously examined treatment rates for people with or at risk of osteoporosis and fracture. A 2011 study of 821 older men and women showed more than 90% were not prescribed treatment before their fracture, while treatment rates increased from 11% to 50% between 2005 and 2008 among older adults admitted with an osteoporotic fracture.^20^ A follow-on study by these authors noted prescribing rates were lower in a rural setting at 39%, and a concerning finding of 1-year adherence of less than 50%.^21^ Another study of both prescribing omissions and inappropriate prescribing showed lower rates among 115 older Irish participants seen in primary care clinics than those of the Netherlands and Switzerland. Of particular note, the authors show prescribing omission rates of less than 2% for osteoporosis medication and less than 4% for calcium and vitamin D prescriptions.^10^ A much larger study of 44 general practices and more than 40 000 primary care patients across multiple practices notes 12.2% were taking a ‘bone health’ prescription at baseline. During follow-up, 2992 patients were initiated on osteoporosis medication, mostly by their GP, but only 21% of 8193 with osteoporosis or at high risk of fracture. Importantly, the authors note 43.6% of those initiated on treatment were deemed lower risk for fracture or did not have a diagnosis of osteoporosis.^22^ These findings are more in line with our results, suggesting the problem is more prevalent when a larger breadth of practice is taken into account. A persistence rate of just above 50% was noted at 2 years.^22^ Finally, a recent study of treatment rates among 3798 women from 8 European countries found the treatment gap was lowest for Ireland at 53% compared with a mean of 75% among those deemed at increased risk of fracture. In this study, a diagnosis of osteoporosis was associated with an 11-fold greater rate of treatment among those deemed at risk, while less than 10% of those not at risk or without a diagnosis of osteoporosis were treated.^23^

A particularly striking finding in our study is the widespread prescribing of calcium and vitamin D, which is almost twice the rate of prescribing of osteoporosis medication in the DXA group irrespective of treatment indication. Although a French study demonstrated residential dwelling adults with a mean age of 84 years had a significant reduction in fractures when treated with calcium and vitamin D,^25^ several large trials have subsequently not shown a clear convincing benefit.^26^,^28^ Some leading independent medical guideline groups no longer recommend such intervention among men and women without osteoporosis.^29^ One of the largest clinical trials in postmenopausal women demonstrated that such supplements increased the risk of hip fracture among younger women, increased the risk of nephrolithiasis and gastrointestinal symptoms.^26^ These supplements cost around the same amount annually as an oral bisphosphonate such as alendronate.

### Strengths and limitations

Our study has important strengths and limitations. This is the first large study of a group of patients cared for across a range of services including GP, HC and OS. This cohort represents individuals referred for DXA and therefore does not reflect the general Irish population. However, it is representative of those typically evaluated for osteoporosis risk in routine practice. We have included detailed list of particular risk factors, men and women, and many who have already had a DXA scan and assessment. At the time of DXA scanning, our nurse specialists take time to gather these data correctly, taking particular care to validate details of prior fractures and current osteoporosis treatment. Patients who have had multiple prior fractures and low BMD but are still not on treatment are assessed by our fracture liaison service, but we do not have the resources to manage everyone. Our study does not have a comprehensive or complete dataset, so it is very likely some important information is missing. The high proportion of missing prescribing information in the referral group limits the precision and generalisability of estimates derived from referral data. We did not include other DXA sites such as the lumbar spine, radius 33% or total hip. Because lumbar spine T-scores were unavailable, individuals with osteoporosis isolated to the spine, particularly early postmenopausal women, may have been misclassified as treatment-ineligible. Studies indicate that 10%–20% of women with non-osteoporotic femoral-neck T-scores have lumbar-spine osteoporosis, suggesting that true treatment eligibility in our cohort is likely underestimated. We do not have access to outside radiology in private hospitals, or other records as we lack a national electronic medical record. We do not have access to GP or other records to ascertain why women were prescribed hormone replacement therapy or whether there were other reasons to prescribe calcium and vitamin D. Other studies suggest that many patients are not taking their medication even when it is prescribed. The reasons people who appear to be appropriate candidates for osteoporosis medication are not on treatment are not available, so there may be genuine concerns about safety or contra-indications we are not aware of. The cross-sectional nature of the study does not allow us to assess subsequent prescribing rates or medication cessation rates, but our empirical evidence from running osteoporosis services suggests these problems are extensive and problematic. However, the prescribing pattern we see among those referred for or who have had DXA scans does reflect what we see in clinical practice: prescribing calcium and vitamin D for osteoporosis rather than an osteoporosis medication, and the widespread prescribing of calcium and vitamin D and sometimes osteoporosis medications for men and women who have no indication for any treatment or supplements.

### Policy implications

We have highlighted the treatment gap for Irish adults with osteoporosis today remains unacceptably large. There is widespread prescribing of calcium and vitamin D, which lacks a strong evidence base, and has risks and associated costs. Contemporaneously, there is considerable prescribing for individuals at low risk or without a clear indication for treatment, which also has a considerable cost. The discrepancy between aggregate national estimates and individual-level prescribing patterns may reflect ecological bias, although this cannot be demonstrated within the design of the present study. This study further supports the urgent need to establish a national osteoporosis policy and clinical programme based on accepted standards of clinical practice. This will improve the value of the care provided by promoting quality and accurate prescribing and reducing waste.

## Conclusion

The osteoporosis treatment gap for patients in Ireland today remains unacceptably large. Concurrently, inappropriate prescribing is also a significant problem which must be addressed. Although prior studies suggest Ireland has one of the smallest treatment gaps in Europe, our findings show substantial under-treatment among individuals who meet guideline-based criteria for osteoporosis therapy, alongside notable prescribing among those without treatment indications. These results highlight opportunities to improve identification and management of high-risk patients in Irish clinical practice. A national osteoporosis programme based on standards of care, education and guidance for busy clinicians, and for patients, could greatly enhance the accuracy of care, prescribing and improve quality and value of care without increasing cost.

## Supplementary material

10.1136/bmjopen-2025-107028 online supplemental material 1

## Data Availability

Data are available upon reasonable request.
