# Supplementary material for: Osteoporosis treatment gap and prescribing patterns in Ireland: a cross-sectional analysis of the DXA HIP project
Source: BMJ Open. 2026 Mar 9;16(3):e107028. doi: 10.1136/bmjopen-2025-107028 (PMC12983729; doi:10.1136/bmjopen-2025-107028)
Supplement: online supplemental material 1 [file bmjopen-16-3-s001.docx]

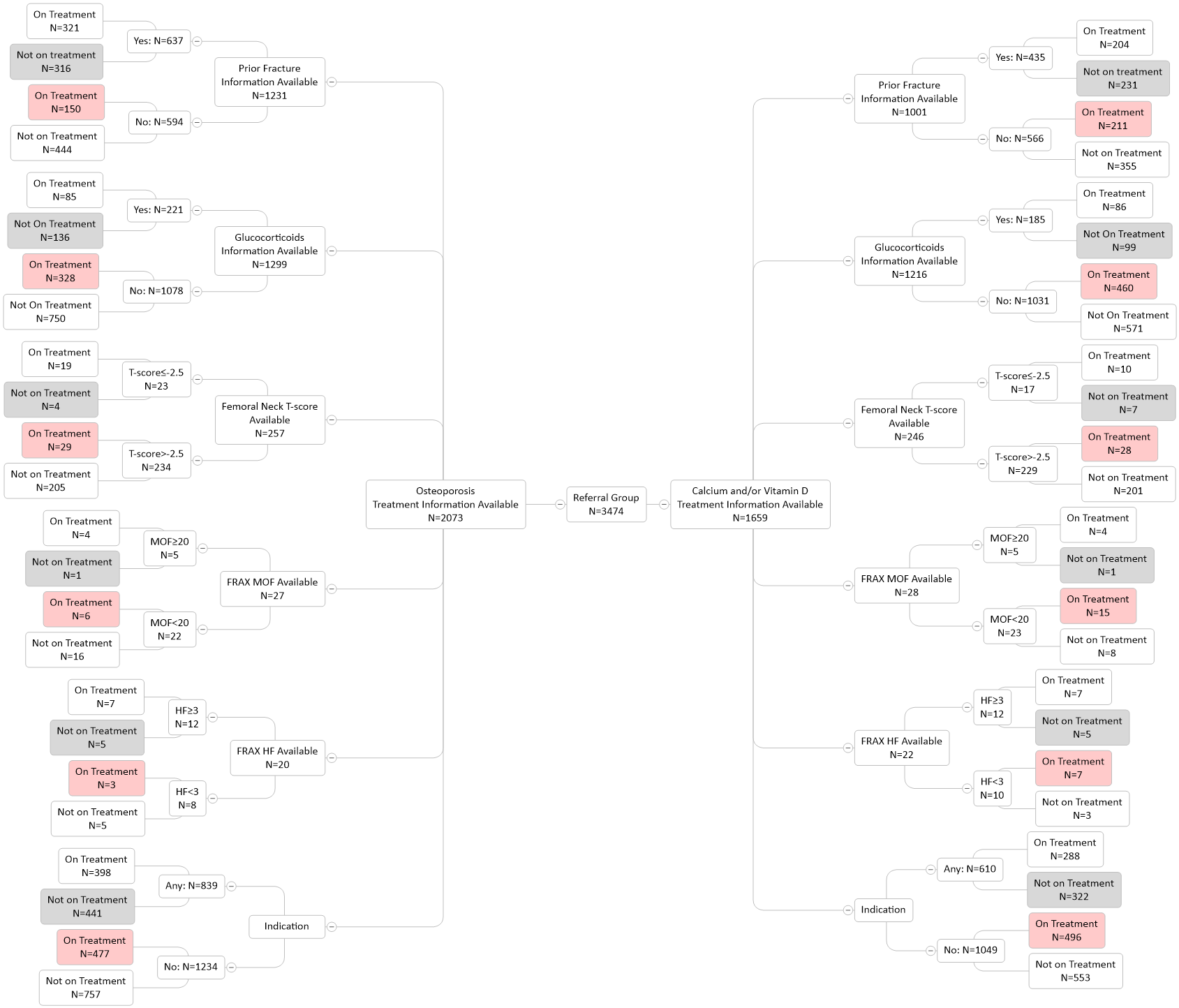


Supplementary Figure 1. Flowchart illustrating patient classification within the referral group based on treatment information availability


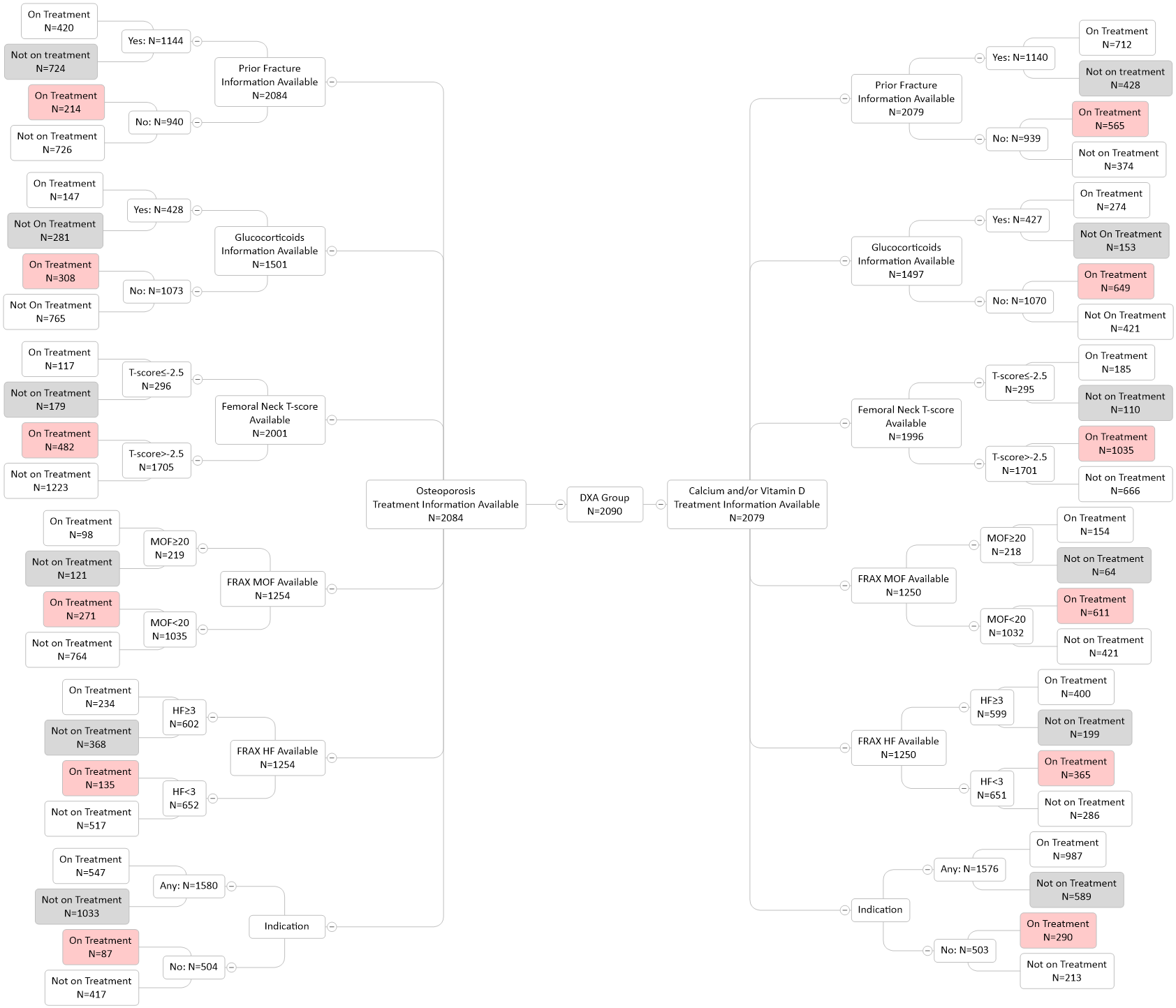


Supplementary Figure 2. Flowchart illustrating patient classification within the DXA group based on treatment information availability
