# Supplementary material for: Osteoporosis treatment gap and prescribing patterns in Ireland: a cross-sectional analysis of the DXA HIP project
Source: BMJ Open. 2026 Mar 9;16(3):e107028. doi: 10.1136/bmjopen-2025-107028 (PMC12983729; doi:10.1136/bmjopen-2025-107028)
Supplement: Supplementary file 2 [file bmjopen-2025-107028.reviewer_comments.pdf]

PEER REVIEW HISTORY

BMJ Open publishes all reviews undertaken for accepted manuscripts. Reviewers are asked to complete a checklist review form and are provided with free text boxes to elaborate on their assessment. These free text comments are reproduced below.

ARTICLE DETAILS

Title (Provisional)

Osteoporosis Treatment Gap and Prescribing Patterns in Ireland: A Cross-Sectional Analysis of the DXA HIP Project

Authors

Carey, John J; Brennan, Attracta; Armstrong, Catherine; Heaney, Fiona; Dempsey, Aoife; Egan, Rebecca; Gorham, Kelly; Yang, Lan

---

VERSION 1 - REVIEW

**Reviewer**	**1**
**Name**	**Makras , Polyzois**
**Affiliation**	**Hellenic Air Force**
**Date**	**20-Jul-2025**
**COI**	**None**

The manuscript addresses a critical issue in osteoporosis care—the apparent paradox of low treatment gap reports for Ireland despite clinical impressions of under- and over-treatment. The study leverages real-world data from DXA referrals to interrogate the validity of ecological assessments. However, there are serious ambiguities in the definitions and metrics used, specifically regarding the concept of “indication for treatment,” the interpretation of calcium/vitamin D prescribing, and the inclusion of estrogen therapy in the treatment analysis. Without resolving these methodological and definitional concerns, the conclusions risk misinterpretation or overstatement.

Major Comments

1. The definition of "Indication for Treatment" is Vague and Inconsistently Applied

Issue: The term "appropriate indication of treatment" is used extensively (e.g., section 3.2) but is never clearly operationalized. It is unclear whether the same criteria were applied across all patient subsets (e.g., prior fracture, FRAX score, T-score ≤ -2.5, glucocorticoid use). This creates confusion in interpreting Tables 2–5, where stratified data are presented by indication.

The authors must define precisely and up front how a patient was classified as having an “indication for treatment.”

2. Use of Calcium and Vitamin D (Ca/D) Prescriptions is Misleading Without Context

Issue: The manuscript uses Ca/D prescriptions to assess under- and over-treatment, but Ca/D is also commonly prescribed for non-osteoporotic reasons (e.g., osteopenia, secondary hyperparathyroidism, or even preventive care in older adults). In section 3.2 and especially the quoted sentence: "Among those with at least 1 indication for treatment, 41.7% were not prescribed calcium and vitamin D..." It’s unclear whether Ca/D use was assumed to be necessary in all indicated cases or if it was a surrogate marker for broader treatment. Authors should clarify the clinical rationale for analyzing Ca/D prescriptions separately and in parallel with osteoporosis medications. Additionally, provide exact figures on how many patients were:

Prescribed both Ca/D and osteoporosis meds

Prescribed only Ca/D

Prescribed neither

3. Estrogen Therapy Inclusion May Skew Treatment Interpretation. Estrogen is included among osteoporosis medications, but its use for bone health is no longer standard in many international guidelines and is often prescribed for menopausal symptoms. Including estrogen prescriptions risks overestimating treatment rates and misrepresenting prescribing appropriateness. I would suggest to remove or separately analyze estrogen therapy. If retained, authors must justify its inclusion with age stratification and clear diagnostic linkage to osteoporosis (e.g., was BMD available for these patients?).

4. Lack of Lumbar Spine DXA Consideration is a Serious Limitation, although it is already stated by the authors. The analysis uses only femoral neck T-scores, while lumbar spine DXA is often more sensitive for detecting osteoporosis in early or postmenopausal women. This limitation likely led to misclassification of patients as not having an indication when they may in fact have met treatment thresholds based on spine scores. Authors must clearly acknowledge this limitation in both Methods and Discussion, with an estimate or sensitivity analysis (if possible) on how this might have affected treatment gap classification.

Minor Issues:

-Abstract: Clearly define what “appropriate prescribing” means and specify the criteria used.

-Figures/Tables: Consider adding a supplementary flowchart to show patient classification (e.g., indication → treated vs. not).

-Terminology: The phrase “appropriate indication” is tautological and should be avoided.

-FRAX application: Clarify if FRAX was used among patients already on treatment.

**Reviewer**	**2**
**Name**	**Premaor, Melissa**
**Affiliation**	**Universidade Federal de Santa Maria**
**Date**	**19-Oct-2025**
**COI**	**None**

The present study is a cross-sectional analysis of two subgroups of a previously described convenience cohort (The DXA HIP Project). The authors explore the osteoporosis treatment GAP in Ireland.

1) Abstract – the conclusion is confusing. How does the "data suggest the majority of patients with osteoporosis or at high risk of fracture in Ireland today DO NOT receive appropriate osteoporosis treatment, while inappropriate prescribing is substantial." and also suggest an ecological fallacy has created a false reassurance of the osteoporosis TREATMENT GAP in Ireland?

2) Methods – How representative of the Irish population is the DXA HIP Project?

3) Outcomes – please clarify that treatment-eligible people were the ones presenting the "following commonly agreed intervention thresholds [15–17]" and all else were treatment-ineligible.

4) Outcomes – Please give a clear definition of calcium and vitamin D eligibility.

5) Outcomes – Please clarify the "indication for treatment" and "the appropriate indication for treatment"

6) Results – Why were the patients who underwent DXA analyzed separately from the referrals? How do these two subgroups differ?

How do the referral subjects that have the treatment information missing differ from the ones who don't?

7) Results – Why is the inappropriately prescribed medication somewhat reassuring?

8) Results – The appropriate statistical analysis for tables 2 to 5 would not be reclassification?

9) Discussion, Strengths and limitations – please add how representative of the Irish population the DXA HIP Project is.

10) Discussion, Strengths and limitations – Please add how the 59.7% and 47.8% missing information about calcium/vitamin D or medication would affect the generalizability of the results.

11) Conclusion – The conclusion should relate to the data presented in the results. The authors do not compare the rates of treatment in Ireland and other countries in this study;

therefore, they cannot conclude that there is an ecological fallacy. The ecological fallacy can be a hypothesis addressed in the discussion. It needs to be better supported for the clarity of the reader.

VERSION 1 - AUTHOR RESPONSE

Reviewer: 1

Dr. Polyzois Makras , Hellenic Air Force

Major Comments

1. The definition of "Indication for Treatment" is Vague and Inconsistently Applied

Issue: The term "appropriate indication of treatment" is used extensively (e.g., section 3.2) but is never clearly operationalized. It is unclear whether the same criteria were applied across all patient subsets (e.g., prior fracture, FRAX score, T-score ≤ -2.5, glucocorticoid use). This creates confusion in interpreting Tables 2–5, where stratified data are presented by indication.

The authors must define precisely and up front how a patient was classified as having an "indication for treatment."

Response:

We now define treatment eligibility explicitly and consistently at the start of the Outcomes section, using four internationally recognised intervention thresholds.

Added:

"Treatment eligibility was defined as at least one of the following criteria: prior fracture, femoral-neck T-score ≤ –2.5, glucocorticoid use, or a FRAX major osteoporotic fracture risk ≥20% or hip fracture risk ≥3%."

2. Use of Calcium and Vitamin D (Ca/D) Prescriptions is Misleading Without Context

Issue: The manuscript uses Ca/D prescriptions to assess under- and over-treatment, but Ca/D is also commonly prescribed for non-osteoporotic reasons (e.g., osteopenia, secondary hyperparathyroidism, or even preventive care in older adults). In section 3.2 and especially the quoted sentence: "Among those with at least 1 indication for treatment, 41.7% were not prescribed calcium and vitamin D..." It's unclear whether Ca/D use was assumed to be necessary in all indicated cases or if it was a surrogate marker for broader treatment. Authors should clarify the clinical rationale for analyzing Ca/D prescriptions separately and in parallel with osteoporosis medications. Additionally, provide exact figures on how many patients were:
Prescribed both Ca/D and osteoporosis meds

Prescribed only Ca/D
Prescribed neither

Response:

Thank you for this comment. Calcium and vitamin D supplementation are recommended for "all patients" in some guidelines, but only if necessary by others. What we see in clinical practice, not here, all the time, are doctors prescribing, and patients taking, calcium and vitamin D as a "treatment" for osteoporosis, which it isn't, and not taking osteoporosis medication. This "myth" persists and is very prevalent (we see patients from around the country, and some from Europe and North America). The largest studies of calcium and vitamin D among community dwelling postmenopausal women shows no overall benefit for fracture reduction (WHI, NEJM 2007)_and there are side effects. We did not attempt to analyse other reasons, but used osteoporosis treatment indications as outlined for both supplementation and osteoporosis medication use. We have added this limitation to our discussion section. The required figures are as follows.

Referral Group:

- Prescribed both Ca/D and osteoporosis meds: 374
- Prescribed only Ca/D: 373
- Prescribed neither: 785

DXA Group:

- Prescribed both Ca/D and osteoporosis meds: 508
- Prescribed only Ca/D: 769
- Prescribed neither: 679

3. Estrogen Therapy Inclusion May Skew Treatment Interpretation. Estrogen is included among osteoporosis medications, but its use for bone health is no longer standard in many international guidelines and is often prescribed for menopausal symptoms. Including estrogen prescriptions risks overestimating treatment rates and misrepresenting prescribing appropriateness. I would suggest to remove or separately analyze estrogen therapy. If retained, authors must justify its inclusion with age stratification and clear diagnostic linkage to osteoporosis (e.g., was BMD available for these patients?).

Response:

Thank you for this comment. We agree that while oestrogen therapy is no longer prescribed for osteoporosis by bone physicians, in Ireland there is a national campaign and policy in place for prescribing hormone replacement therapy for several years and it is now 'free' of charge. As it is one of the reasons listed for a DXA scan by referring primary care doctors we believe it is important to be transparent. However the indications for this therapy are not on the referral forms. Generally we would not add or recommend additional anti-resorptive therapy if a patient is already on oestrogen therapy in our clinical practice. We appreciate opinions differ on this, as do clinical guidelines, particularly between endocrinologists, gynaecologists, primary care doctors and our service (rheumatology). Thus, we believe it would be inappropriate to

exclude this as a ‘treatment’ even if it is not something we recommend. We have acknowledged this limitation in the discussion section.

4. Lack of Lumbar Spine DXA Consideration is a Serious Limitation, although it is already stated by the authors. The analysis uses only femoral neck T-scores, while lumbar spine DXA is often more sensitive for detecting osteoporosis in early or postmenopausal women. This limitation likely led to misclassification of patients as not having an indication when they may in fact have met treatment thresholds based on spine scores. Authors must clearly acknowledge this limitation in both Methods and Discussion, with an estimate or sensitivity analysis (if possible) on how this might have affected treatment gap classification.

Response:

We thank the reviewer for emphasising the importance of lumbar spine BMD. We agree that using femoral-neck T-scores alone may misclassify some individuals, particularly early postmenopausal women who often show discordance with more severe osteoporosis at the spine. Although lumbar spine measurements were not available within the DXA HIP dataset, we have now strengthened the Methods and Discussion to explicitly acknowledge this limitation.

A formal sensitivity analysis is not possible because spine values were not recorded; however, we note that population-level studies report lumbar-spine osteoporosis in approximately 10–20% of women who have non-osteoporotic femoral-neck T-scores. We now reference this in the Discussion to illustrate the potential direction and magnitude of misclassification.

Added the following sentence in Method:

“Only femoral-neck T-scores were available in this dataset. Lumbar spine BMD measurements were not recorded, and therefore individuals with osteoporosis isolated to the spine could not be identified.”

Added this as a separate limitation in Discussion:

“Because lumbar spine T-scores were unavailable, individuals with osteoporosis isolated to the spine, particularly early postmenopausal women, may have been misclassified as treatment-ineligible. Studies indicate that 10–20% of women with non-osteoporotic femoral-neck T-scores have lumbar-spine osteoporosis, suggesting that true treatment eligibility in our cohort is likely underestimated.”

Minor Issues:

-Abstract: Clearly define what “appropriate prescribing” means and specify the criteria used.

Response:

We agree the term required precise definition. The abstract now explicitly defines

treatment eligibility using recognised intervention thresholds and clarifies how prescribing appropriateness was assessed.

Added sentence:

"Treatment eligibility was defined using established intervention thresholds, including prior fracture, femoral-neck T-score ≤-2.5, glucocorticoid use, or FRAX major osteoporotic fracture risk ≥20% or hip fracture risk ≥3%."

-Figures/Tables: Consider adding a supplementary flowchart to show patient classification (e.g., indication → treated vs. not).

Response:

We confirm that two flowcharts have been added and referenced in the text.

-Terminology: The phrase "appropriate indication" is tautological and should be avoided.

Response:

All instances of "appropriate indication" were replaced with the standard terms treatment-eligible and treatment-ineligible defined explicitly in the Methods.

-FRAX application: Clarify if FRAX was used among patients already on treatment.

Response:

Clarified that FRAX values were derived using femoral-neck BMD, consistent with FRAX guidance.

Reviewer: 2

Dr. Melissa Premaor, Universidade Federal de Santa Maria

1) Abstract – the conclusion is confusing. How does the "data suggest the majority of patients with osteoporosis or at high risk of fracture in Ireland today DO NOT receive appropriate osteoporosis treatment, while inappropriate prescribing is substantial." and also suggest an ecological fallacy has created a false reassurance of the osteoporosis TREATMENT GAP in Ireland?

Response:

We revised the conclusion to align strictly with the study's findings and removed the implication. Instead, we state that aggregate estimates may underestimate the true treatment gap.

Replaced with:

“These findings suggest that the true treatment gap in Ireland is substantially larger than aggregate estimates imply.”

2) Methods – How representative of the Irish population is the DXA HIP Project?

Response:

We added a statement clarifying representativeness and the expected referral bias due to clinical indications for DXA testing.

Added:

“This cohort represents individuals referred for DXA based on clinical indications. It reflects the population typically evaluated for osteoporosis risk in routine clinical practice.”

3) Outcomes – please clarify that treatment-eligible people were the ones presenting the "following commonly agreed intervention thresholds [15–17]" and all else were treatment-ineligible.

Response:

Added explicit classification rules.

Inserted:

“Individuals meeting any of the above thresholds were classified as treatment-eligible. All others were classified as treatment-ineligible.”

4) Outcomes – Please give a clear definition of calcium and vitamin D eligibility.

Inserted definition:

“Dietary calcium intake questionnaires and biochemical tests for vitamin D, calcium, phosphate and PTH are performed in outpatient clinics to help determine the need for such supplements but not available for these cohorts from the DXA registry. In clinical practice we find the majority of those taking supplements do not need them, while many of those who do are not.”

5) Outcomes – Please clarify the "indication for treatment" and "the appropriate indication for treatment"

Response:

These terms have been replaced with the standard framework of “treatment-eligible” and “treatment-ineligible” based on explicit thresholds. The manuscript now uses only those terms.

Inserted definition:

“Treatment eligibility was defined as meeting at least one of the above criteria: prior fracture, femoral-neck T-score ≤ –2.5, glucocorticoid use, or a FRAX major osteoporotic fracture risk ≥20% or hip fracture risk ≥3%.”

6) Results – Why were the patients who underwent DXA analyzed separately from the referrals? How do these two subgroups differ? How do the referral subjects that have the treatment information missing differ from the ones who don't?

Response:

We agree this required clarification. We added a concise explanation. The Referral Group reflects real-world referrals and contains substantial missing prescribing information. The DXA Group includes patients with validated medication histories collected by trained nurses, providing near-complete treatment data. Thus, analysing them separately prevents misclassification due to differing data completeness.

Added:

“The referral and DXA groups were analysed separately because prescribing information was missing in up to 59.7% of referrals but was almost complete in the DXA group, where medication details were verified at the time of scanning. Analysing the groups separately reduces misclassification resulting from differences in data completeness.”

7) Results – Why is the inappropriately prescribed medication somewhat reassuring?

Response: Thank you. This sentence should have included the word ‘only’.

8) Results – The appropriate statistical analysis for tables 2 to 5 would not be reclassification?

Response:

We clarified that the tables aim to describe proportions treated vs untreated within eligibility categories, not to perform reclassification analysis. The one-proportion Z-test was used only to compare the proportion treated vs not treated.

Added sentence:

“The analyses were descriptive and did not constitute a reclassification analysis. One-proportion Z-tests were used solely to compare the proportion prescribed treatment versus not prescribed treatment within each eligibility criterion.”

9) Discussion, Strengths and limitations – please add how representative of the Irish population the DXA HIP Project is.

Response:

We updated the limitations section to state clearly that the population reflects referred individuals rather than the wider population.

Added:

"This cohort represents individuals referred for DXA and therefore does not reflect the general Irish population. However, it is representative of those typically evaluated for osteoporosis risk in routine practice."

10) Discussion, Strengths and limitations – Please add how the 59.7% and 47.8% missing information about calcium/vitamin D or medication would affect the generalizability of the results.

Response:

Added explicit comment that high missingness limits inference on referral prescribing patterns.

Added:

"The high proportion of missing prescribing information in the referral group limits the precision and generalisability of estimates derived from referral data."

11) Conclusion – The conclusion should relate to the data presented in the results. The authors do not compare the rates of treatment in Ireland and other countries in this study; therefore, they cannot conclude that there is an ecological fallacy. The ecological fallacy can be a hypothesis addressed in the discussion. It needs to be better supported for the clarity of the reader.

Response:

We thank the reviewer for this clarification. We agree that our study does not include cross-country analyses and therefore cannot establish the presence of ecological fallacy. Our intention was to highlight a potential inconsistency between aggregate national estimates and individual-level treatment patterns.

To address this, we have removed the claim of ecological fallacy from the Conclusion and now present it only as a possible explanation discussed in the Interpretation section. We also clarify that ecological bias cannot be demonstrated within the design of our study.

Change in Discussion:

Added:

“The discrepancy between aggregate national estimates and individual-level prescribing patterns may reflect ecological bias, although this cannot be demonstrated within the design of the present study.”

Change in Conclusion:

“Our findings show substantial under-treatment among individuals who meet guideline-based criteria for osteoporosis therapy, alongside notable prescribing among those without treatment indications. These results highlight opportunities to improve identification and management of high-risk patients in Irish clinical practice.”

VERSION 2 - REVIEW

**Reviewer**	**1**
**Name**	**Makras , Polyzois**
**Affiliation**	**Hellenic Air Force**
**Date**	**03-Dec-2025**
**COI**	

The authors have adequately addressed almost all raised issues.

Regarding the minor comment: "Clarify if FRAX was used among patients already on treatment" I meant that authors should clearly state that FRAX calculation was applied only among non-treated patients. Otherwise FRAX cannot be applied in a patient already on treatment.

VERSION 2 - AUTHOR RESPONSE

Reviewer: 1

Dr. Polyzois Makras , Hellenic Air Force

Major Comments

The authors have adequately addressed almost all raised issues.

Regarding the minor comment: "Clarify if FRAX was used among patients already on treatment" I meant that authors should clearly state that FRAX calculation was applied only among non-treated patients. Otherwise FRAX cannot be applied in a patient already on treatment.

Response:

We thank the reviewer for this important clarification. We agree that FRAX is not validated for use in patients already receiving osteoporosis treatment. We have now revised the Methods section to explicitly state that FRAX calculations were applied only to individuals who were not receiving osteoporosis medication at the time of referral or DXA assessment.

Added:

"FRAX scores were calculated only for individuals who were not receiving osteoporosis medication at the time of referral or DXA assessment, as FRAX is not validated for patients already on treatment."
